# The Enhancement of the Thermal Conductivity of Epoxy Resin Reinforced by Bromo-Oxybismuth

**DOI:** 10.3390/polym15234616

**Published:** 2023-12-04

**Authors:** Yuan Jia, Beibei Li, Huan Ma, Juxiang Yang, Zhen Liu

**Affiliations:** The Key Laboratory for Surface Engineering and Remanufacturing in Shaanxi Province, College of Chemical Engineering, Xi’an University, Xi’an 710065, China; beibeili0813@xawl.edu.cn (B.L.); 15769202603@163.com (H.M.); jxyang05@xawl.edu.cn (J.Y.); liuz@xawl.edu.cn (Z.L.)

**Keywords:** thermal conductivity, dielectric properties, epoxy, bromo-oxygen-bismuth

## Abstract

With the gradual miniaturization of electronic devices, the thermal conductivity of electronic components is increasingly required. Epoxy (EP) resins are easy to process, exhibit excellent electrical insulation properties, and are light in weight and low in cost, making them the preferred material for thermal management applications. In order to endow EPs with better dielectric and thermal conductivity properties, bromo-oxygen-bismuth (BiOBr) prepared using the hydrothermal method was used as a filler to obtain BiOBr/EP composites, and the effect of BiOBr addition on the properties of the BiOBr/EP composites was also studied. The results showed that the addition of a small amount of BiOBr could greatly optimize the dielectric properties and thermal conductivity of EP resin, and when the content of BiOBr was 0.75 wt% and 1.00 wt%, the dielectric properties and thermal conductivity of the composite could reach the optimum, respectively. The high dielectric constant and excellent thermal conductivity of BiOBr/EP composites are mainly due to the good layered structure of BiOBr, which can provide good interfacial polarization and thermal conductivity.

## 1. Introduction

With the rapid development of electronic technology, all kinds of electronic products are developing towards high integration, high frequency, and diversified functions; there is a growing demand for microelectronic capacitors, which not only need a low thickness, but also put forward very strict requirements for a low dielectric loss and high thermal conductivity [1,2]. Most of the traditional capacitors are made of inorganic materials. Although capacitors made of inorganic materials have the advantages of a very high dielectric constant and good thermal stability, their preparation process is complex, the materials are too brittle, and the dielectric loss is too large, which greatly limits their application in the field of high-tech electronics [3]. In addition, microelectronic capacitors also put forward higher requirements for the miniaturization and integration of dielectric materials, and the rapid decline of their thickness even approaches the physical limit of materials. Therefore, traditional inorganic materials cannot meet the requirements of capacitors in the field of high-tech electronics [4]. Therefore, it is necessary to find new materials to give them a good thermal conductivity on the basis of meeting a high dielectric constant and low dielectric loss.

Polymers have the advantages of easy processing, excellent electrical insulation performance, and being light weight and low cost; thus, it is imperative to use polymers as the matrix of dielectric materials [5]. Among them, epoxy resin (EP) has been widely used in the preparation of various electronic and electrical equipment because of its excellent electrical characteristics, good thermal stability and low production cost [6,7]. However, the low dielectric properties of polymer materials limit their application in microelectronic capacitors [8], so it is necessary to add nano-fillers with a high dielectric constant or easy polarization into EP [9], so as to increase the dielectric constant without increasing the dielectric loss. In actual use, the heat resistance of epoxy resin is also very important for its use as an electronic component. The heat resistance of epoxy resin is affected by its molecular skeleton structure, the curing agent used, and the curing process, etc. [10]. At present, there are many methods to improve the heat resistance of epoxy resin at home and abroad, such as: developing epoxy resin with a new heat resistant skeleton structure; synthesising the curing agent of epoxy resin with a new structure; and blending or copolymerization with inorganic nanomaterials [11,12,13]. In addition, the high integration degree of electronic products will cause the heat inside the material to not easily emit, and heat accumulation in the circuit board will reduce the service life of the circuit board to a certain extent. Therefore, while ensuring the excellent dielectric properties of EP composites, it is also necessary to consider the optimization of their thermal conductivity [14]. There are many methods to improve the overall performance of EP, and the simplest option is to directly add high-performance fillers to the polymer matrix [15]. A large number of studies show that, for composites meeting the above requirements, it is necessary to consider the relevant properties of the fillers, among which the structure, morphology, and dielectric properties of the filler are very important [16]. Inorganic materials with excellent thermal conductivity, such as boron nitride, aluminium nitride, and alumina, etc., have already been used in EP to enhance its thermal conductivity. Nano-ceramic fillers have a high thermal conductivity and electrical conductivity and low cost, which can effectively increase the thermal conductivity of epoxy resin [17]. However, nano ceramic filler has limited compatibility with epoxy resin, which easily agglomerates in the epoxy resin matrix and affects its mechanical properties, and its different crystal structures will greatly affect its thermal conductivity [18]. Carbon materials are light in weight, have a high strength and good corrosion resistance, and are widely used in the field of aerospace [19]. Due to the large number of carbon isomers, different crystal structures will affect the thermal conductivity of carbon materials. Metal and its oxides have a good electronic thermal conductivity, so they are often used as epoxy resin modifiers. However, when the amount is too large, it will affect the viscosity of EP to a certain extent. Therefore, it is necessary to find new inorganic fillers to modify EP [20,21]. Compared with circular fillers, linear and flake fillers with a higher specific surface area can better improve the thermal conductivity of composites [22]. However, the problems such as being fragile, easy aggregation, and poor fluidity would limit the application effect of this kind of fillers [23]; therefore, how to apply linear and flaky thermal conductive fillers to polymer matrix composites has become one of the most concerned problems. 

BiOBr is a PbCl-type structure in which internal atoms are connected by strong covalent bonds and the atomic layers are held together by weak van der Waals forces [24], forming a layered structure that is separated along the (001) direction [25]. This open-crystal structure creates enough space to facilitate the polarization of atoms and related orbitals [26]. Because the dielectric property of a material refers to its polarization property under the action of an applied electric field, the higher the polarization strength, the greater the corresponding dielectric constant [27]. Therefore, the high polarization effect of BiOBr is beneficial for improving the dielectric constant of the resin composite. At the same time, its good lamellar structure can greatly reduce the percolation threshold of composite materials, which can ensure that the thermal conductivity of composite materials can be greatly improved when the filling amount is low [28]. In addition, BiOBr has very stable chemical properties, enabling it to maintain its original electronic structure and physical properties during the preparation process [29,30]. Thus it can be predicted that the addition of BiOBr to EP resin can effectively improve the thermal conductivity of epoxy resin while improving its dielectric constant. In addition, BiOBr with a closed structure can not only maintain the original layered structure and original excellent properties of BiOBr, but also endow it with more stable physical properties, so as to ensure that BiOBr can maintain the original morphology during the preparation of EP composites. At the same time, the excellent properties of BiOBr itself enable the BiOBr addition of a small amount in the EP matrix to greatly optimize the dielectric properties and thermal conductivity of the composites, without causing aggregation in the composites or affecting the properties of the EP itself. Therefore, this study intends to choose bismuth bromo-oxybromide as a filler to optimize the thermal conductivity of EP. 

In summary, this study firstly prepared BiOBr with a closed structure via the hydrothermal method and added it into EP resin as an inorganic modification component. BiOBr/EP composites with different contents of BiOBr were then obtained using the curing method. The effects of the BiOBr addition amount on the dielectric constant, dielectric loss, and thermal conductivity of EP resin were also studied. The results showed that, when the content of BiOBr was 0.75 wt% and 1.00 wt%, the dielectric constant and thermal conductivity of the BiOBr/EP composites reached their maximum values, respectively, and the dielectric loss was still within the acceptable range. Meanwhile, the heat resistance of the BiOBr/EP composite was optimized to some extent by adding BiOBr. This is due to the good polarizability of BiOBr under an applied electric field and the high thermal conductivity and layered structure of BiOBr. This study lays a theoretical foundation for the application of BiOBr in the preparation of resin EP composites with a high dielectric constant and high thermal conductivity.

## 2. Experimental Methods

### 2.1. Materials

The raw materials for preparing BiOBr are: Bi(NO_3_)_3_·5H_2_O (AR, the purity of >99.0%, Shanghai Aladdin Biochemical Technology Co., Ltd., Shanghai, China) and KBr (AR, the purity of >99.0%, Tianjin Beichen Founder Reagent Factory, Tianjin, China). The EP resins (E-51 typed, CP, the purity of >95 wt%, the epoxy equivalent is 184–194 g/mol, the viscosity is 12,000–16,000 mPa·s, the hydrolyzed chlorine content is 0.25–0.65%, the moisture content ≤ 0.04%, and the volatile matter content ≤ 0.3%) were obtained from Nantong Xingchen Synthetic Material Co. Ltd., Nantong, China. Isophorone diamine (AR, the purity of >99.0%, Shanghai McLean Biochemical Technology Co., Ltd., Shanghai, China) was used as a curing agent. The other reagents and solvents used in this research without further purification were: experiments including: glycol and anhydrous ethanol (AR, the purity of >99.0%, Tianjin Fuchen Chemical Reagents Factory, Tianjin, China).

### 2.2. Preparation of the BiOBr

In total, 0.9675 g of Bi(NO_3_)_3_·5H_2_O was dissolved in 100 mL of glycol and stirred with magnetic stirrer for 30 min to obtain a 0.02 mol/L Bi(NO_3_)_3_ solution. A total of 0.0714 g of KBr was dissolved in a 30 mL Bi(NO_3_)_3_ solution, and stirred at room temperature for 20 min, the mixture was then poured into a teflon-lined reactor, sealed, and placed in an oven at 160 °C for 12 h. After natural cooling to room temperature, the obtained precipitation was transferred into a 50 mL centrifuge tube and centrifuged with deionized water and anhydrous ethanol 3 times each. The centrifuge rate was set at 8000 r/min and the centrifuge time was set at 10 min. The centrifuged sample was placed in a vacuum drying oven and dried at 60 °C for 12 h. The powder obtained was BiOBr.

### 2.3. Preparation of the BiOBr/EP Composites

**Figure 1:**
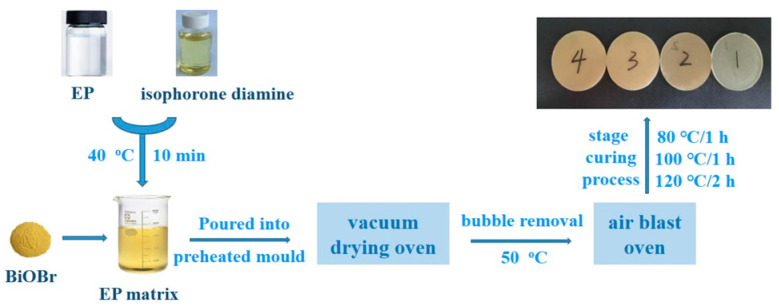
The preparation process of BiOBr/EP composites.

The BiOBr obtained above was used as a filler to add into the EP matrix with different additions to prepare the BiOBr/EP composites via a casting method. The specific preparation process was as follows: the EPs were stirred in a glass beaker with isophorone diamine at 40 °C for 10 min to prepare theEP matrix, and the BiOBr (the mass ratios of BiOBr to epoxy resin were 0.00 wt%, 0.25 wt%, 0.50 wt%, 0.75 wt%, and 1.00 wt%, respectively) was added into the EP matrix. After 15 min of ultrasonic dispersion of these mixtures, the BiOBr/EP pre-polymer with well-dispersed BiOBr was obtained, which was then poured into the preheated mould. The mould was placed in a vacuum drying oven at 50 °C and bubbles were pumped for about 30 min to ensure that there were no bubbles or defects inside the composites. Finally, the mould containing the BiOBr/EP pre-polymer was put into the air blast oven, and the BiOBr/EP composites were obtained through the process of segmenting curing. The curing process was as follows: 80 °C/1 h + 100 °C/1 h + 120 °C/2 h. The preparation process is shown in Figure 1. The size of the BiOBr/EP composites was about 52 ± 1 mm in diameter and about 4 ± 1 mm in thickness.

### 2.4. Measurements

#### 2.4.1. The X-ray Diffraction (XRD)

The crystal structure of BiOBr was researched using X-ray diffraction (XRD, Bruker D8, Karlsruhe, Germany) at room temperature.

#### 2.4.2. Scanning Electron Microscopy (SEM)

The morphology of BiOBr and the fractured surface features of the BiOBr/EP composites were observed using a scanning electron microscope (SEM, HITACHI, Tokyo, Japan) at room temperature.

#### 2.4.3. Dielectric Properties

The dielectric properties of the BiOBr/EP composites were measured with a dielectric constant dielectric loss tester (ZJD-A type, China Aviation Times Company, Beijing, China) at room temperature. Five samples were selected for each different BiOBr content, each sample was tested three times, and the average was selected after measurement. 

#### 2.4.4. Thermal Conductivity

The thermal conductivity coefficient of the BiOBr/EP composites was measured with a thermal conductivity tester (KDRX-Ⅱ, Xiangtan Xiangyi Instrument Co., Ltd., Xiangtan, China) via the transient fast hot wire method. Five samples were selected for each different BiOBr content, and the average was selected after measurement.

#### 2.4.5. Thermal Resistant Properties

The thermogravimetric analysis (TGA) of BiOBr/EP was researched under a nitrogen atmosphere with the TGAQ50, and the heating rate was 20 °C min^−1^. 

## 3. Results and Discussion

### 3.1. Morphology Structure of the BiOBr

**Figure 2:**
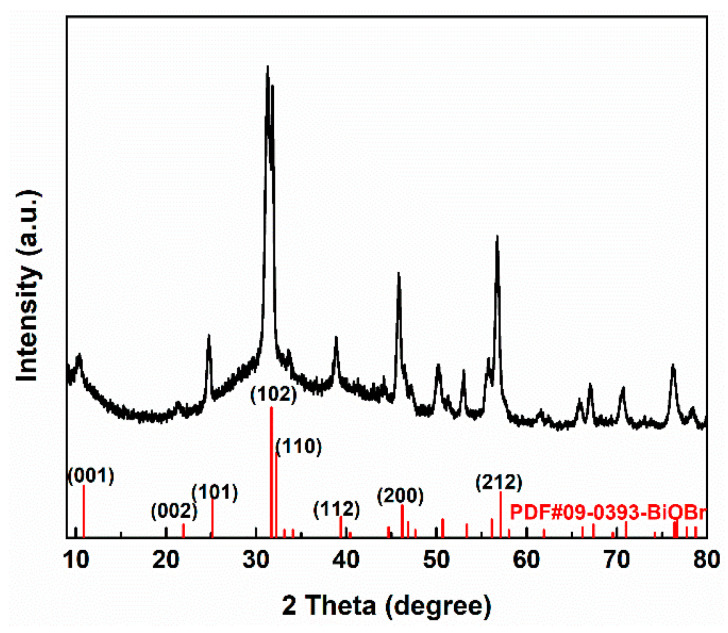
The XRD result of BiOBr.

**Figure 3:**
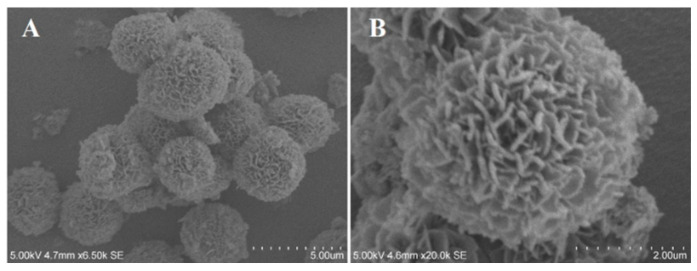
The SEM results of BiOBr ((**A**) Surface structure of BiOBr microspheres; (**B**) Surface structure of BiOBr microspheres 2.5 times larger than A).

The crystal structure of BiOBr was researched using X-ray diffraction (XRD), and the surface morphology of BiOBr was observed using scanning electron microscopy (SEM); the results are shown in Figure 2 and Figure 3, respectively. It can be seen from Figure 2 that the crystallinity of BiOBr obtained with the solvothermal treatment was strong. The characteristic peaks of BiOBr were located at 10.9, 25.1, 31.7, 32.2 45.7, and 56.8°, corresponding to the (001), (101), (102), (110), (200), and (212) crystalline facets, which were in good agreement with the tetragonal phase of BiOBr (JCPDS93-0393).

Figure 3 shows the micrograph of the BiOBr microspheres with 3–5 μm diameters that were successfully obtained on a large scale after the solvothermal treatment at 160 °C for 12 h. The exterior surfaces of the microspheres are not clearly smooth, but contain an extensive growth of sheet-like structures with thickness around 100 nm.

### 3.2. Dielectric Properties of BiOBr/EP

**Figure 4:**
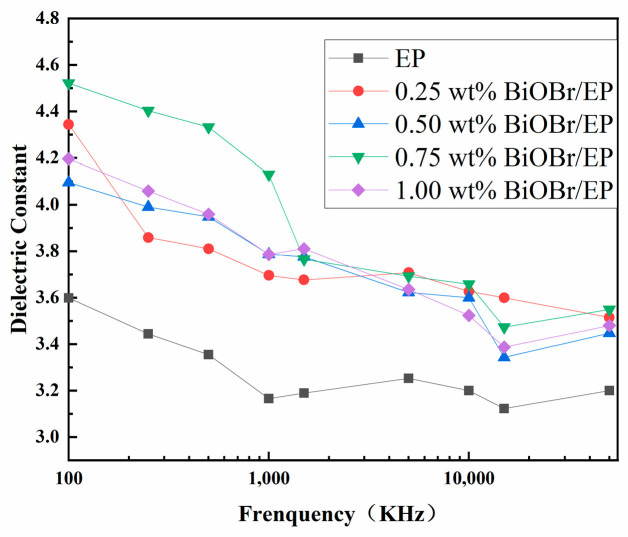
The dielectric permittivity of BiOBr/EP with different content of BiOBr varies by frequency.

The dielectric constant of the BiOBr/EP composites with different contents and structures of BiOBr were researched, and the result is shown in Figure 4. As can be seen from Figure 4, the dielectric constant of the BiOBr/EP composite increased to a greater extent than that of EP, even with a small amount of BiOBr being added to EP, and the dielectric constant of the BiOBr/EP composites gradually and steadily increased with the increase in BiOBr content in the range from 0.25 wt% to 0.75 wt%. The reason can be attributed to the semiconductivity of BiOBr, while the dielectric constant of the interface layer is small, and the volume of the interface region is larger than that of the material; thus, the interface layer plays a dominant role in the reduction in the dielectric constant of the material. However, when some conductive particles were introduced into the EP matrix, polarization occurred in two phases, resulting in a significant increase in the dielectric constant of the BiOBr/EP composites. Therefore, with the increase in the BiOBr amount, the number of interfaces between the fillers and the resin matrix increased, and the polarization of the interface was further enhanced, resulting in an increase in the dielectric constant of the BiOBr/EP composites. In addition, when the content of BiOBr reached 1.00 wt%, the dielectric constant of the BiOBr/EP composites decreased slightly compared to that of BiOBr/EP with 0.75 wt% BiOBr. The main reason may have been that the leakage current of the BiOBr/EP composites under a high filling volume was larger, which made the charge storage capacity of the composite begin to decline. At the same time, when the content of BiOBr in the BiOBr/EP composites was very high, part of the BiOBr was reunited together, which could not achieve the effect of enhancing the dielectric properties of the BiOBr/EP composites, thus increasing the polar group matrix and steric hindrance, resulting in a decrease in the dielectric constant of the BiOBr/EP composites. It can also be observed from the figure that the dielectric constants of BiOBr/EP composites with different contents of BiOBr all showed a decreasing trend with an increase in the frequency, showing the law of a high dielectric constant at a low frequency. This phenomenon can be attributed to an increase in frequency and an increase in the electric field change speed. The polarization of polar groups in the EP composites lagged behind the change in the electric field, and the polarization order was as follows: the first was the surface polarization process, then the orientation polarization process, and finally, the displacement polarization process. Thus, the effect of polarization on the dielectric constant of the BiOBr/EP composites decreased significantly. 

**Figure 5:**
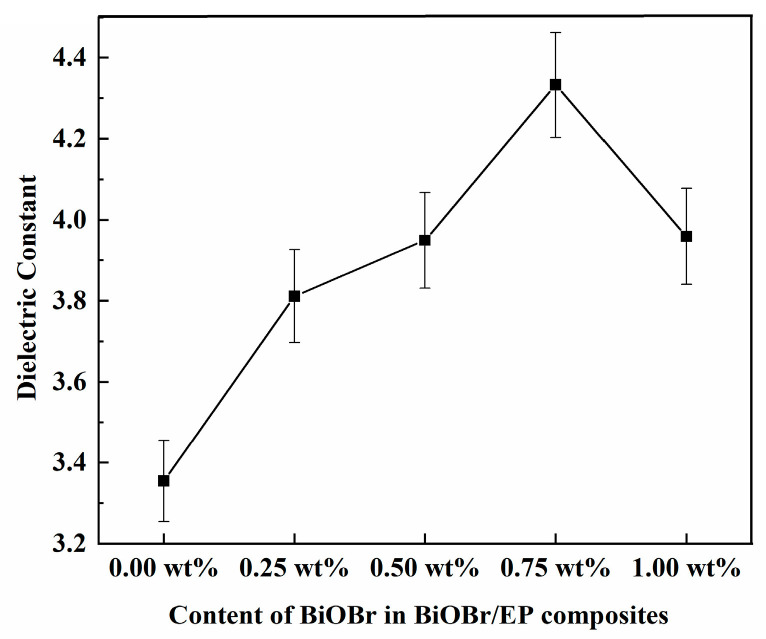
The dielectric constants of BiOBr/EP varies with the contents of S-BiOBr at 5 MHz.

**Figure 6:**
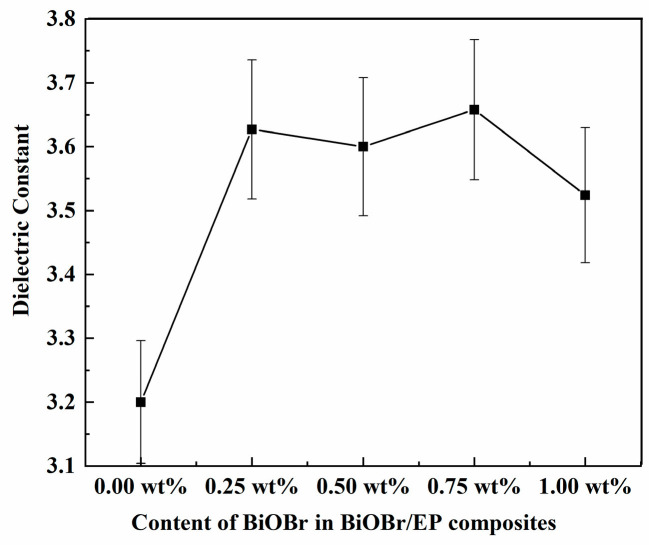
The dielectric permittivity of BiOBr/EP under 10 MHz.

Figure 5 and Figure 6 show that the dielectric constants of BiOBr/EP varied with the contents of BiOBr under 500 kHz and 10 MHz, respectively. As can be seen from Figure 5, under the low-frequency electric field, the dielectric constant of the BiOBr/EP composite material increased first and then decreased with an increase in BiOBr content, and reached the maximum (4.333) when the content was 0.75 wt%, and then began to decrease with further BiOBr content being added. This is because, under a low-frequency electric field, the addition of an appropriate content of BiOBr can effectively improve the dielectric property of the material, but when the content is too high, excessive BiOBr begins to aggregate, and the contact between them will affect the surface polarization effect, resulting in a decrease in the dielectric constant. Under the high-frequency electric field (10 MHz, as shown in Figure 6), although the dielectric constant of the BiOBr/EP composites also increased to a certain extent after the addition of the BiOBr, there was no obvious dependence on the amount of BiOBr added. This was because the relaxation polarization, such as the interface polarization of the BiOBr, was not established in time under the high-frequency electric field, so the effect of the BiOBr addition amount on the dielectric constant of the BiOBr/EP composites was not obvious.

**Figure 7:**
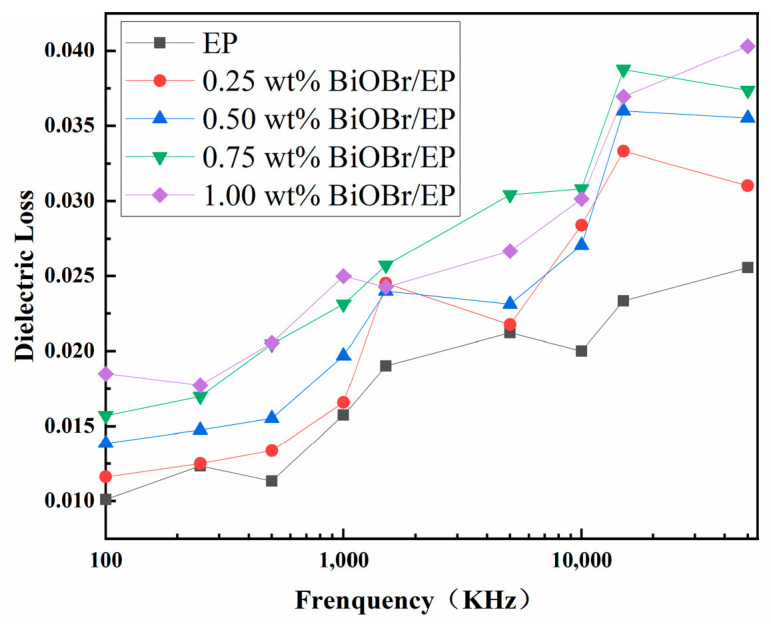
The dielectric loss of BiOBr/EP with different content of BiOBr varies with frequency.

The dielectric loss of the BiOBr/EP composites changing with the frequency is shown in Figure 7. As can be seen from the figure, compared to pure EP, the dielectric loss of the BiOBr/EP resin composites showed an increasing trend. This was because, after adding BiOBr into the resin, the interface polarization introduced into the material led to a certain increase in dielectric loss. When the frequency was low, the polarization velocity of the dipole inside the composite material followed the change velocity of the upper electric field, and this process consumed less energy. However, when the frequency was too high, the polarization of the dipole could not keep up with the change in the electric field. At this time, part of the energy needed to be absorbed to overcome the friction resistance. Therefore, more losses were generated at a high frequency. However, in general, the maximum dielectric loss was also within the controllable range (≤0.05), which can meet the requirements of high dielectric properties in the field of microelectronic components.

**Figure 8:**
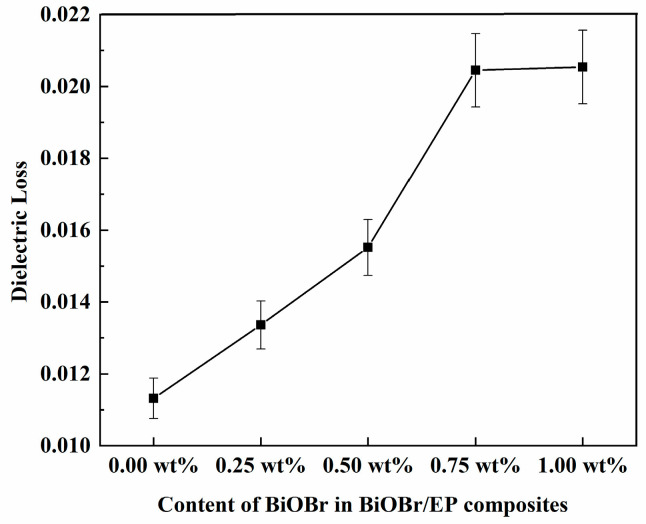
The dielectric loss of BiOBr/EP under 500 kHz.

**Figure 9:**
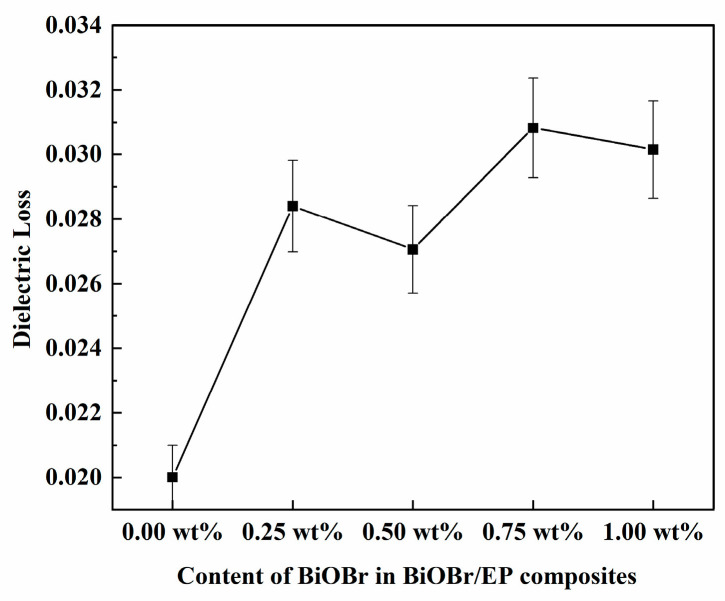
The dielectric loss of BiOBr/EP under 10 MHz.

Figure 8 and Figure 9 are the dielectric losses of the BiOBr/EP composites with different contents of BiOBr at 500 kHz and 10 MHz, respectively. As can be seen from Figure 9, under the low-frequency electric field (500 kHz), with a gradual increase in BiOBr content, the dielectric loss of the BiOBr/EP composites presented a state of first a sharp increase and then stable, and the maximum dielectric loss was 0.02045 with 0.75 wt% BiOBr. However, under the high-frequency electric field (10 MHz), the dielectric loss of the BiOBr/EP composite increased after adding BiOBr, but the increase in the dielectric loss had little dependence on the content of the BiOBr, and the highest dielectric loss was 0.03082 with 0.75 wt% BiOBr. In addition, although the dielectric loss increased, this was still within the usable range. This is because, in a low-frequency electric field, the addition of BiOBr can enhance the interface polarization, so its content had a great effect on the dielectric loss of the BiOBr/EP composite. When BiOBr content was too high, part of the BiOBr would aggregate, its polarization effect decreased, and the dielectric loss gradually became stable. However, in the case of the high-frequency electric field, when the polarization in the medium could not keep up with the change in the external electric field, the dielectric loss increased, but its dependence on the BiOBr content was weakened.

### 3.3. Thermal Conductivities of the Materials

**Figure 10:**
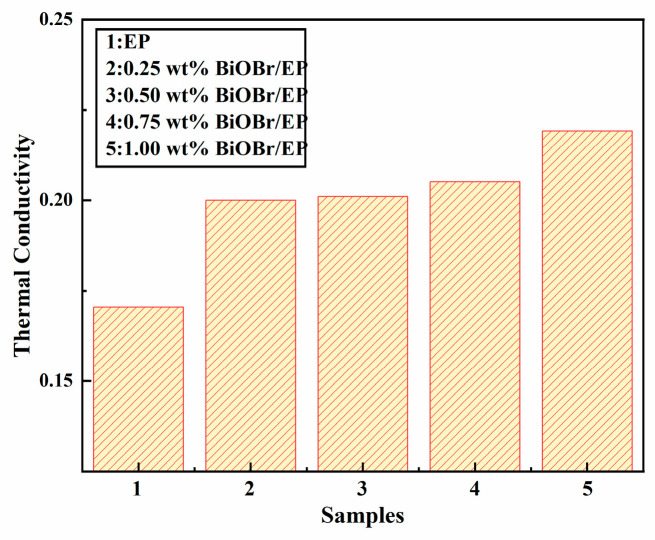
The thermal conductivities of BiOBr/EP with different content of BiOBr.

Figure 10 shows the thermal conductivity of the BiOBr/EP composites with different contents of BiOBr. As can be seen from the figure, when the BiOBr/EP composites containing BiOBr were added, their thermal conductivity was higher than that of pure EP (0.1705 W/mk), and increased with an increase in BiOBr content. This was because, when the BiOBr content was small, the filler was isolated in the polymer, resulting in a large spacing between the fillers and no contact with each other, and it was difficult to form a continuous thermal conductivity channel. This is equivalent to the packing particles being coated by polymer, and the fillers being bridged by polymer; thus, the improvement in the thermal conductivity of the composite materials was limited. With the increase in the BiOBr amount, the thermal resistance of the interface between the BiOBr and the EP matrix was improved, the thermal conductivity pathway was established to form the thermal conductivity network and improve the heat transfer efficiency, and the thermal conductivity increased accordingly. When the content of BiOBr was 1.00 wt%, the thermal conductivity of BiOBr/EP could be up to 0.2190 W/mk, which was 28.45% higher than that of pure EP (0.1705 W/mk). Our team has prepared two kinds of spherical MoS_2_ with different structures to enhance the thermal conductivity of EP; the results show that, when the addition amount of molybdenum disulfide was 3.0 wt%, the thermal conductivity of these two MoS_2_/EP composites reached the maximum value, which was 0.3061 W/mK and 0.3105 W/mK, respectively [31]. However, when the addition amount of BiOBr prepared in this study was only 1.0 wt%, the thermal conductivity of EP could be increased to 0.2190 W/mK, indicating that the thermal conductivity of EP could be better optimized even if a small amount of BiOBr was added, which can be owed to the layered structure of BiOBr that can better perform thermal conductivity in EP resin.

**Figure 11:**
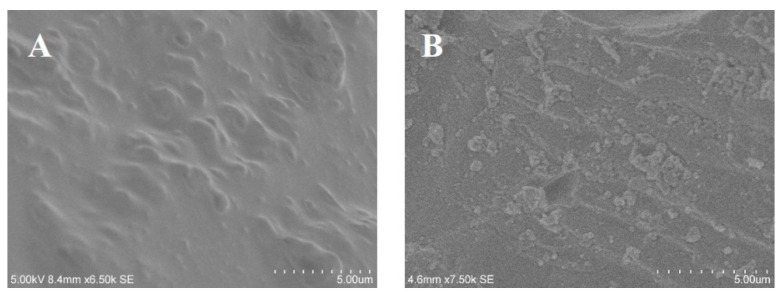
SEM of fracture surfaces taken from the BiOBr/EP composites ((**A**): EP; (**B**): BiOBr/EP composites with 0.75 wt% BiOBr).

**Figure 12:**
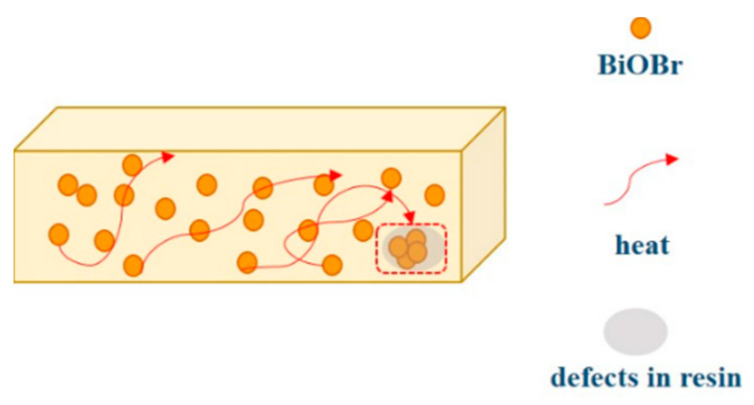
Heat conduction mechanism of EP with BiOBr.

In order to further study the thermal conductivity mechanism in the composites, EP and the fracture surface of the BiOBr/EP composites were observed. The results are shown in Figure 11. As can be seen from Figure 11A, the surface of the EP resin was smooth, although some bumps appeared, but this was due to the interpenetrating structure generated by the molecular chains inside the EP resin, which provided a way for heat transfer. As for the BiOBr/EP composite, there were very obvious small particles on its surface. Most of these particles were isolated from each other, and a few of them were in contact with each other, which provided a good thermal conduction path for heat transfer. Therefore, the transfer of heat inside the BiOBr/EP composite not only depended on the molecular chain of the EP resin, but also through the BiOBr. BiOBr particles are small and have a high thermal conductivity, so a small amount of addition could effectively improve the thermal conductivity of the composite. However, after excessive BiOBr was added to the EP resin, agglomeration would occur, resulting in the formation of a cavity in the material, resulting in the partial aggregation of heat; therefore, the appropriate content of BiOBr was beneficial for the thermal conductivity of the BiOBr/EP composites (as shown in Figure 12).

### 3.4. Thermal Resistant of the Materials

**Figure 13:**
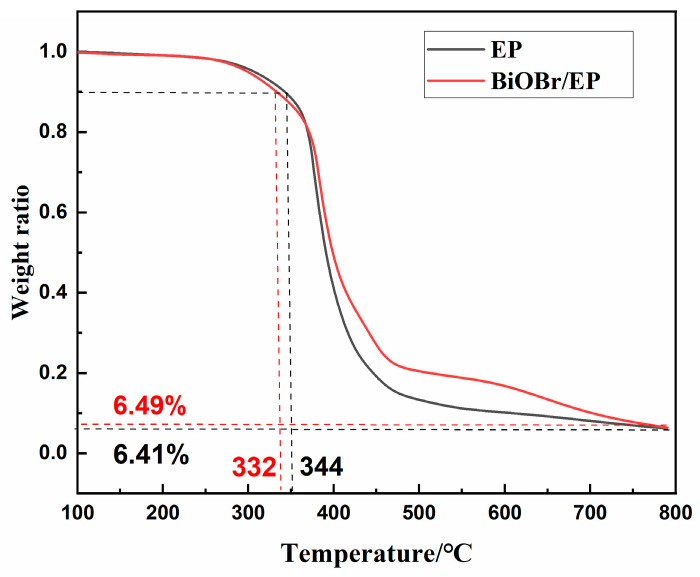
The TGA curve of the EP and BiOBr/EP composites with 0.75 wt% BiOBr.

Figure 13 shows the TGA curve of pure EP and the BiOBr/EP (BiOBr: 0.75 wt%) composites. As can be seen from the figure, the initial decomposition temperature (the temperature when the mass loss rate is 5%) of the BiOBr/EP composite was 302 °C, and that of pure EP was 310 °C, indicating that the addition of BiOBr improved the initial thermal stability of EP to some extent. When the temperature continued to rise to 800 °C, the residual carbon rate of BiOBr/EP was 6.49%, which was not much different from that of pure EP resin (6.41%). However, within the temperature range of 400–700 °C, the residual carbon rate of the BiOBr/EP composite was always much higher than that of the pure EP, indicating that BiOBr/EP had a better thermal stability. The improved thermal stability of the BiOBr/EP composite was not only because the Bi and halogen Br atoms in BiOBr/EP structure can greatly improve the heat resistance of materials, but also because of the synergistic effect between these atoms. In addition, the structure of BiOBr can provide a part of the molecular cavity to meet the heat transfer inside the resin. At the same time, both of the BiOBr/EP composites and the EP could form a uniform and stable interpenetrating network structure after copolymerization; thus, excellent resistance to external heat damage. In addition, isoflurone diamine was selected as the curing agent in the preparation of BiOBr/EP material, and there was also a heat-resistant synergistic effect between the molecules, which made BiOBr/EP have a better heat resistance.

**Figure 14:**
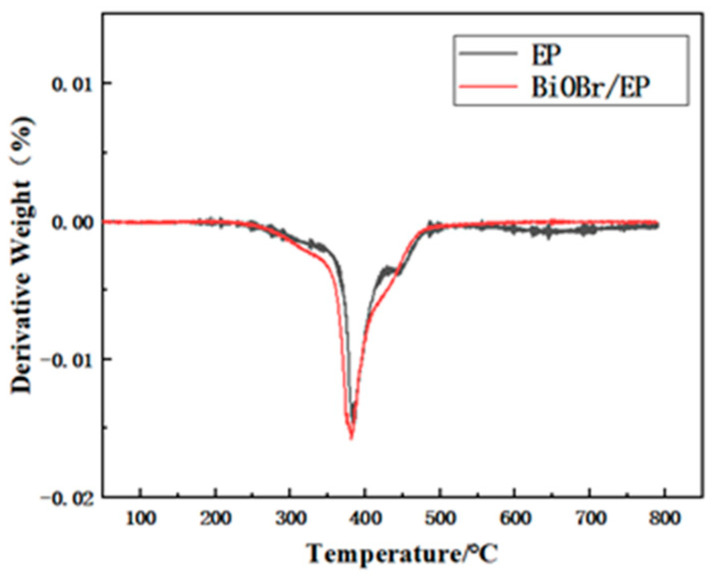
The DTG curve of the EP and BiOBr/EP composites with 0.75 wt% BiOBr.

Figure 14 shows the DTG curves of the pure EP and BiOBr/EP (BiOBr: 0.75 wt%) composites. It can be seen from Figure 14 that the curves of the pure EP and BiOBr/EP composites were roughly the same, which indicates that the addition of BiOBr did not change its decomposition mechanism.

## 4. Conclusions

In this study, spheroidal BiOBr with a layered structure was prepared via the hydrothermal method and used as filler to modify EP resin. The results showed that the dielectric constant and dielectric loss of the BiOBr/EP composites exhibited a good stability and applicability under a low-frequency electric field when the BiOBr addition amount was 0.75 wt%, and the thermal conductivity of the composite could be as high as 0.2190 W/mk when the BiOBr additive amount was 1.00 wt%, which was 28.45% higher than that of pure EP (0.1705 W/mk). The improvement in the dielectric and thermal conductivity of the BiOBr/EP composites can be attributed not only to the good size effect and dispersion of BiOBr, but also to the excellent thermal conductivity and interfacial polarization of BiOBr itself. Meanwhile, the heat resistance of the BiOBr/EP composite was also optimized, and this was because the existence of Br atom itself would improve the heat resistance of the composites, while isoflurone diamine was selected as the curing agent, and there was also a good heat resistance synergistic effect between molecules. This study provides a new idea for the preparation of EP resin with a high thermal conductivity.

## Data Availability

Data are contained within the article.
